# The Effects of a Pre-workout Supplement on Measures of Alertness, Mood, and Lower-Extremity Power

**DOI:** 10.7759/cureus.24877

**Published:** 2022-05-10

**Authors:** Jason Curtis, Cassandra Evans, Veronica Mekhail, Paulina Czartoryski, Juan Carlos Santana, Jose Antonio

**Affiliations:** 1 Exercise and Sports Science, Keiser University, West Palm Beach, USA; 2 Nutrition/Exercise and Sports Sciences, Rocky Mountain University of Health Professions, Ft. Lauderdale, USA; 3 Nutrition/Exercise and Sports Sciences, Nova Southeastern University, Ft. Lauderdale, USA; 4 Fitness and Performance, Institute of Human Performance, Boca Raton, USA; 5 College of Health Care Sciences, Nova Southeastern University, Davie, USA

**Keywords:** performance, exercise, focus, supplement, caffeine

## Abstract

Objective

The purpose of this study was to investigate the effects of a multi-ingredient pre-workout supplement (MIPS) on mental and physical performance.

Materials and methods

Fourteen exercise-trained men (n=7) and women (n=7) completed this randomized, placebo-controlled, double-blind, counterbalanced, crossover trial. Participants consumed either a MIPS or a placebo in a randomized, counterbalanced order. Forty-five minutes after consumption, the following assessments were conducted: psychomotor vigilance test (PVT), Profile of Mood States (POMS), vertical jump test, and heart rate and blood pressure. There was a one-week washout period between assessments.

Results

Statistically significant differences were observed between the treatment and placebo groups for the PVT (reaction time: treatment: 286 ± 28 ms, placebo: 306 ± 46 ms, p=0.0371) and POMS (i.e., vigor: treatment: 15.2 ± 14.9, placebo: 9.7 ± 9.6, p=0.0403; fatigue: treatment: 1.0 ± 1.1, placebo: 3.3 ± 3.4, p=0.0100). There were no significant differences between groups for the other indices of mood, false starts from the PVT, and vertical jump.

Conclusion

Based on our findings, the acute consumption of a MIPS produced a significant improvement in a sustained-attention, reaction-timed task as well as measures of vigor and fatigue.

## Introduction

Multi-ingredient pre-workout supplements (MIPS) are commonly marketed as “pre-workouts” and have been shown to positively affect mental and physical performance [1-6]. Even though the primary ingredient in most MIPS is caffeine (CAF) [1-6], a multitude of other ingredients is also found in these supplements. In active females, the acute ingestion of MIPS has been shown to improve factors such as focus, anaerobic capacity, and upper body muscular endurance following high-intensity exercise [7]. Energy drinks, which are often consumed prior to training, have demonstrated an improved time to exhaustion as well as improvement in terms of subjective measures of energy, fatigue, and focus [6,8]. A study that examined the effects of a CAF-containing energy drink versus a placebo found that those who consumed the energy drinks had fewer false starts during a psychomotor vigilance test (PVT), suggesting it elicited some improved performance in PVT testing [9].

Other ingredients found in MIPS may also favorably affect mental and physical performance [e.g., tyrosine, theanine, L-citrulline, alpha-glycerophosphocholine (alpha-GPC)]. Cutrufello et al. have explored the effects of a single dose of L-citrulline prior to exercise [6]. Subjects consumed one of three beverages (L-citrulline, watermelon juice, or placebo) one or two hours prior to exercise, and it was found that the single dose of L-citrulline did not have any effect on exercise performance [10]. Creatine supplementation has been shown to enhance cognitive functioning and work as a neuroprotective supplement [higher levels of creatine reduced injuries to the brain and spinal cord due to planned traumatic brain injuries (TBIs) in rat studies] [11]; however, this is due to chronic (daily use) not acute (single occurrence) consumption. Every MIPS has different blends of primary ingredients; thus, it is difficult to make a direct comparison between different MIPS. The MIPS studied in the current investigation consisted of several potentially ergogenic ingredients including CAF, L-citrulline, betaine, beta-alanine, creatine, and alpha-GPC. Different combinations of ingredients in MIPS may produce different results vis-à-vis the mental and physical performance. In light of this, the purpose of this study was to investigate the effects of MIPS on mental and physical performance.

## Materials and methods

Participants

Fourteen exercise-trained men (n=7; mean age: 19.9 ± 1.4 years) and women (n=7; mean age 19.9 ± 1.4 years) volunteered for this randomized, placebo-controlled, double-blind, counterbalanced, crossover trial. Subjects reported to the lab on two separate occasions a week apart to participate in testing. In accordance with the Declaration of Helsinki, the Institutional Review Board for Keiser University approved all procedures involving human subjects prior to the beginning of the data collection (IRB# IRB000SP21JC101, Keiser University). This study was approved through an expedited review in February 2021. All participants signed a written informed consent that was approved by the Institutional Review Board of the university (IRB# IRB000SP21JC101) prior to conducting any study-related activities. Daily CAF consumption and exercise history were assessed via self-reporting.

Body composition

A multi-frequency bioelectrical impedance device (InBody 270) was used on the participants to assess their body composition on their first visit to the laboratory (body mass, fat mass, lean body mass, body fat percentage, and total body water in liters). Each subject was instructed to arrive after having fasted for at least three hours. Participants stood on the device platform with bare feet on the electrodes. They were then instructed by the device to grasp the handles (which contain additional electrodes on the thumb and fingers) while they kept their arms straight and horizontally adducted at approximately 30 degrees. This assessment was completed within approximately one minute.

Multi-ingredient pre-workout supplement (MIPS)

The MIPS and placebo were donated by Shifted, LLC (Eugene, OR). The placebo comprised maltodextrin. Both the placebo and the MIPS were placed in identical bags with different codes. Both powders were identical in appearance and taste and were mixed with ~8-12 fluid ounces of water in opaque water bottles for consumption. Table 1 lays out the nutritional facts panel of the Shifted MIPS.

**Table 1:** Nutritional facts panel of the Shifted MIPS *Percentage DV is based on a 2,000-calorie diet. **DV not established DV: daily value; MIPS: multi-ingredient pre-workout supplement

Supplement facts		
Serving size: 1 scoop (30 g)		
Servings per container: 20		
	Amount per serving	% DV*
Calories	5	
Total carbohydrate	1 g	<1%
Niacin (as nicotic acid)	15 mg	94%
Vitamin B6 (as pyridoxine HCL)	1 mg	59%
Vitamin B12 (as methylcobalamin)	100 mg	4167%
Iron	1 mg	6%
Magnesium (red spinach leaf extract and dimagnesium malate)	9 mg	2%
Sodium (as pink Himalayan sea salt)	40 mg	2%
Potassium (from red spinach leaf extract and potassium chloride)	248 mg	5%
L-citrulline	8 g	**
Creatine monohydrate	5 g	**
Taurine	3 g	**
Beta-alanine (as Carnosyn®)	2.5 g	**
Betaine anhydrous	2.5 g	**
L-tyrosine	2 g	**
Red spinach leaf extract (as Oxystorm®)	1 g	**
Beetroot extract	1 g	**
Alpha-GPC (50%)	300 mg	**
Caffeine blend	300 mg	**
Caffeine anhydrous (250 mg)		**
zümXR® Delayed-Release Caffeine (50 mg)		**
L-theanine	150 mg	**
ElevATP® (ancient peat and apple fruit extract)	150 mg	**
Pink Himalayan sea salt	100 mg	**
AstraGin® [astragalus membranaceus (root) extract and Panax notoginseng (root) extract]	25 mg	**
BioPerine® (black pepper fruit extract)	5 mg	**
Other ingredients: citric acid, natural flavor, malic acid, silicon dioxide, calcium silicate, sucralose, spirulina powder (color)		

Testing procedures

Participants arrived at the lab on two separate occasions separated by one week between the hours of 1200 and 1400. The subjects read and signed the consent form instructing them to not change dietary or exercise habits during the treatment period. The subjects’ physical characteristics were only assessed on the first visit; during both visits, they filled out the Profile of Mood States (POMS) questionnaire and performed the five-minute PVT and a three-trial max vertical jump test using a vertec. The subjects either consumed the MIPS drink or the placebo (every odd-number subject received the powder from bags with the first code, and every even-number subject received powder from the bags with the second code). On the second visit, they received a coded bag of powder that they had not received on the first visit. The research team was blinded to the treatment product and the placebo, and the company that had sent the bags of MIPS or placebo did not release the information of the codes to the research team until all trials were complete. Forty-five minutes post-consumption (during which all participants were instructed not to leave the lab or consume any food or drink), the subjects filled out the POMS questionnaire and performed the five-minute PVT and the three-trial vertical jump, and then the post-test heart rate (HR) and blood pressure (BP) were measured after all other tests.

Profile of Mood States (POMS)

The POMS is a 65-word standard validated psychological test to assess transient and distinct mood states [12]. The test listed words such as “angry”, “tense”, “lively”, and 62 other words commonly used to describe different mood states. Next to each word is a drop-down menu with the words “how I have felt” above them. The options in the drop-down menu were “not at all”, “a little”, “moderately”, “quite a bit”, and “extremely”. This test scores total mood disturbance, anger, depression, fatigue, tension, and vigor. Those participants who have lower scores would be categorized as having more stable mood profiles [12].

Psychomotor vigilance test (PVT)

A PVT was administered using standard electronic tablets (Apple iPads; Apple Inc., Cupertino, CA) and automated testing software (Vigilance Buddy by Research Buddies). The PVT test displays stimuli (i.e., a number on the screen). As soon as the participants saw the number appear in the middle of the screen, they then touched the screen as fast as they could to get the lowest possible time. Each participant was given instructions on how the test works, and how to properly do the test. The subjects were instructed to respond as fast as possible to the stimulus while not responding prematurely [13]. The PVT assesses vigilant attention and activates the visual cortex, motor cortex, and prefrontal cortex [14].

Statistical analysis

The GraphPad Prism 6 statistical software (GraphPad Software Inc., San Diego, CA) was used to perform all statistical analyses. All data for this study is presented as mean ± standard deviation (SD). A paired t-test was performed to determine whether statistically significant differences (p<0.05) existed between the treatment and the placebo (i.e., for PVT and vertical jump); a Wilcoxon matched-pairs signed-rank test was performed (due to the types of data we analyzed) to determine if differences existed for the various mood assessments.

## Results

The descriptive characteristics (Table 2) of the subjects were as follows: 14 total exercise-trained individuals, men (n=7) and women (n=7) (mean ± SD: age: 19.9 ± 1.4 years; height: 168.2 ± 11.3 cm; body mass: 68.9 ± 10.7 kg). CAF consumption for the treatment was approximately 4 mg per kg based on the mean body mass of the subjects.

**Table 2:** Physical characteristics N=14, seven males, seven females SD: standard deviation (SD)

Variables	Values (mean ± SD)
Age (years)	19.9 ± 1.4
Height (cm)	168.2 ± 11.3
Body mass (kg)	68.9 ± 10.7
Lean body mass (kg)	54.9 ± 12.5
Fat mass (kg)	14.0 ± 5.5
% body fat	21.0 ± 9.0
Total body water (liters)	40.2 ± 9.2
Total number of years of training	6.3 ± 3.5
Average hours of aerobic exercise per week	3.6 ± 3.5
Average hours of resistance exercise per week	7.4 ± 4.9
Other exercises per week (exercises other than resistance training or aerobic training)	1.8 ± 2.8
Average caffeine consumed per day	207 ± 112

HR and BP were measured after testing at 45-minute post-consumption (Table 3), and the results showed no differences between the treatment and the placebo.

**Table 3:** Heart rate and blood pressure Heart rate and blood pressure were assessed 45-min post-consumption of the treatment and placebo. There were no significant differences between the groups except for diastolic BP, which was higher in the placebo group BP: blood pressure; SD: standard deviation

	Treatment, mean ± SD	Placebo, mean ± SD	P-value
Heart rate (bpm)	80 ± 15	79 ± 14	0.8947
Systolic BP (mmHg)	138 ± 15	136 ± 19	0.6696
Diastolic BP (mmHg)	80 ± 11	87 ± 16	0.0077

The results of the POMS (Table 4) are as follows: total mood disturbance score (TMDS): treatment: 1.1 ± 1.5, placebo: 6.2 ± 7.1, p=0.1912; anger: treatment: 2.0 ± 2.0, placebo: 1.4 ± 1.5, p=0.4257; confusion: treatment: 3.4 ± 3.6, placebo: 4.3 ± 4.5, p=0.1386; depression: treatment: 1.2 ± 1.3, placebo: 1.1 ± 1.1, p=0.7701; fatigue: treatment: 1.0 ± 1.1, placebo: 3.3 ± 3.4, p=0.0100; tension: treatment: 8.6 ± 8.4, placebo: 5.9 ± 6.1, p=0.1345; vigor: treatment: 15.2 ± 14.9, placebo: 9.7 ± 9.6, p=0.0403.

**Table 4:** Profile of Mood States (POMS) Self-reported fatigue and vigor were significantly lower and higher, respectively, in the treatment group vs. the placebo TMDS: total mood disturbance score; SD: standard deviation

	Treatment (mean ± SD)	Placebo (mean ± SD)	P-value
TMDS	1.1 ± 1.5	6.2 ± 7.1	0.1912
Anger	2.0 ± 2.0	1.4 ± 1.5	0.4257
Confusion	3.4 ± 3.6	4.3 ± 4.5	0.1386
Depression	1.2 ± 1.3	1.1 ± 1.1	0.7701
Fatigue	1.0 ± 1.1	3.3 ± 3.4	0.0100
Tension	8.6 ± 8.4	5.9 ± 6.1	0.1345
Vigor	15.2 ± 14.9	9.7 ± 9.6	0.0403

The results of the PVT (Table 5) and vertical jump test (Table 5) were as follows: reaction time: treatment: 286 ± 28 ms, placebo: 306 ± 46 ms, p=0.0371; number of false starts: treatment: 3.1 ± 2.4, placebo: 2.5 ± 2.8, p=0.4473; vertical jump (cm): treatment: 54 ± 8, placebo: 54 ± 10, p=0.4927.

**Table 5:** Psychomotor vigilance and vertical jump The treatment group had a faster reaction time vs. the placebo SD: standard deviation

	Treatment (mean ± SD)	Placebo (mean ± SD)	P-value
Reaction time (ms)	286 ± 28	306 ± 46	0.0371
Number of false starts	3.1 ± 2.4	2.5 ± 2.8	0.4473
Vertical jump (cm)	54 ± 8	54 ± 10	0.4927

## Discussion

The current study found an improvement in sustained attention (i.e., mean reaction time on the PVT) as well as levels of perceived fatigue (perception of feeling tired) and vigor (perception of feeling energetic) with the consumption of MIPS; however, there was no effect on vertical jump performance. It should be noted that CAF is a common ingredient in both energy drinks and MIPS; however, there are other ingredients that may also play a role vis-à-vis an ergogenic effect (e.g., L-citrulline, tyrosine, alpha-GPC) [15-30]. The strengths of this study are that it was a double-blind, crossover study. Since all participants belonged to both groups, there were no differences associated with subjects belonging to different groups. This study provides additional insights into ergogenic ingredients found in MIPS.

Adenosine receptors in the central nervous receptors (CNS) are the primary targets for antagonism when CAF is consumed [15]. In a study investigating the effects of CAF and vigilance on the Special Forces personnel by McLellan et al., they found that during 27 hours of wakefulness, vigilance was maintained, and running performance improved [17]. Our current investigation found that 45 minutes after the consumption of a CAF-containing MIPS, the reaction time of the treatment group was significantly better than the placebo group. Our investigation used a dose within the normal range of 3-6 mg per kg of body weight; 300 mg of CAF equates to approximately 4 mg of caffeine per kg of body weight in the current study. This shows that in sports, jobs, and other activities that require elevated levels of vigilance, the consumption of MIPS would be a beneficial ergogenic aid. Our study also found significant improvements in vigor and a reduction in fatigue when comparing the POMS scores between the treatment group and the placebo group. This would also endorse the ability to be more vigilant gained by consuming a MIPS product.

The MIPS contained other potentially ergogenic ingredients. L-citrulline is a non-essential amino acid and a precursor for L-arginine. Several studies have observed improvements in exercise performance following supplementation with L-citrulline. For instance, supplementing with 6-8 grams of citrulline malate 40-60 minutes prior to exercise improves muscular endurance (i.e., increased repetitions) [17]. Several clinical trials have found alpha-GPC supplementation to improve cognitive impairments in patients with cerebral disorders such as dementia and Alzheimer’s disease [18]. Other studies have evaluated the effects of alpha-GPC on physical and psychomotor performance. Marcus et al. have reported no significant differences in psychomotor vigilance task results between alpha-GPC (two capsules of either 250 or 500 mg/day for seven days) and placebo groups [16]. Improvements in cognitive function are more likely in individuals with cognitive impairments compared to healthy, exercise-trained individuals [18]. In a recent study, supplementation of 600 mg alpha-GPC for six days showed improvements in lower body force production compared to placebo [20]. Additionally, 600 mg of alpha-GPC administration 90 minutes prior to resistance exercise showed improvements in peak bench press force, but not peak power or rate of force development [21]. These studies suggest that alpha-GPC administration could improve physical performance, but not cognitive performance, in healthy, exercise-trained individuals.

Tyrosine and theanine also show promise as potential ergogenic aids that may enhance cognitive functions. Older adults were found to have higher plasma tyrosine concentrations in a dose-dependent fashion [22]. Interestingly, working memory in older adults was best at a lower dose (i.e., 100 mg per kg body weight) in comparison to higher doses (i.e., 150 and 200 mg per kg body weight) [22]. An investigation by Kahathuduwa et al. found that a 200-mg dose of theanine improved cognitive measures of selective attention [12] with CAF also demonstrating similar effects. Moreover, the combination of caffeine and theanine ostensibly had additive effects on attention [23]. One of the more intriguing studies involved the acute consumption of caffeine, theanine, and tyrosine [24]. Twenty-four male former collegiate athletes completed mental and physical performance assessments using a Makoto Arena testing device that assessed physical as well as mental performance. Compared to the placebo, the supplement combination of CAF, theanine, and tyrosine improved movement accuracy [24].

Rhodiola rosea is another supplement that is purported to enhance cognitive function [25]. Rhodiola is not one compound but a myriad of different compounds that includes flavonoids and phenylethanol (i.e., Spanish sage) [25]. Scholey et al. discovered that a 333-mg dose of sage improved memory in a group of older adults [22]. Tildesley et al. showed that sage improved immediate word recall [23]. Notwithstanding the intriguing data from these trials, it is evident that more research is needed regarding Rhodiola and/or its constituent compounds. Other ingredients found in MIPS that purported to show promise for enhancing physical and cognitive function include creatine monohydrate, beta-alanine, and betaine. However, it would appear that chronic supplementation is required to elicit such an effect with these supplements. Current research suggests that creatine and beta-alanine require a chronic loading phase of approximately four weeks to meaningfully impact body stores of creatine and carnosine, respectively [28]. Although MIPS contained 2500 mg of beta-alanine and 5000 mg of creatine monohydrate, it is unlikely that these ingredients contributed to any of the effects seen in this study due to the necessity of chronic use [29]. An investigation by Borsook et al. reported increased feelings of well-being and decreased feelings of fatigue due to betaine supplementation in a clinical population [4]. These studies suggest that betaine may improve mood. It is plausible to think betaine could contribute to reported improvements in feelings of vigor, and fatigue; however, more research is needed to validate this.

There are several limitations to this study. We observed elevated BP and HR in both placebo and treatment groups with no significant difference between the groups. This could be due to the “White Coat Syndrome” or the fact that we measured these diagnostics immediately after the vertical jump protocols. Additionally, we only had a total of 14 subjects for this study. A study involving a larger sample size would allow us to discern any differences between sexes or find other significant differences in other measures that may result from a greater sampling number. Also, we did not look at the effects of CAF by genotypes. However, the results of this study do support the findings of previous research regarding the effects CAF products have on PVT.

## Conclusions

The acute consumption of MIPS significantly improved sustained attention as well as perceived vigor and fatigue. It is not clear if there is a specific single ingredient (e.g., CAF) that contributed to this improvement or if it was the result of a combination of ingredients (i.e., CAF, theanine, alpha-GPC, L-tyrosine, etc.). Since there are multiple ingredients within the MIPS, it is not possible to determine which ingredient(s) contributed the most to the ergogenic effect. CAF is perhaps the only ingredient that is present in all MIPS; thus, it behooves future researchers to include a CAF-only positive control. Nevertheless, one can sensibly conclude that the MIPS, as shown in the current investigation, may serve to enhance performance in tasks (e.g., driving) or sports (e.g., baseball) that require sustained attention.
