# Correction: The Effects of a Pre-workout Supplement on Measures of Alertness, Mood, and Lower-Extremity Power

**DOI:** 10.7759/cureus.c128

**Published:** 2023-07-10

**Authors:** Jason Curtis, Cassandra Evans, Veronica Mekhail, Paulina Czartoryski, Juan Carlos Santana, Jose Antonio

**Affiliations:** 1 Exercise and Sports Science, Keiser University, West Palm Beach, USA; 2 Nutrition/Exercise and Sports Sciences, Rocky Mountain University of Health Professions, Ft. Lauderdale, USA; 3 Nutrition/Exercise and Sports Sciences, Nova Southeastern University, Ft. Lauderdale, USA; 4 Fitness and Performance, Institute of Human Performance, Boca Raton, USA; 5 College of Health Care Sciences, Nova Southeastern University, Davie, USA

This article has been corrected to fix improperly ordered references. The reference list is now accurate. The authors and journal greatly regret that this error was not identified and fixed prior to publication.

